# Intersectionality, *BRCA* Genetic Testing, and Intrafamilial Communication of Risk: A Qualitative Study

**DOI:** 10.3390/cancers16091766

**Published:** 2024-05-02

**Authors:** Sharlene Hesse-Biber, Memnun Seven, Hannah Shea, Andrew A. Dwyer

**Affiliations:** 1 Department of Sociology, Boston College, Chestnut Hill, MA 02467, USA; sharlene.hesse-biber@bc.edu (S.H.-B.); hcshea99@gmail.com (H.S.); 2 Elaine Marieb College of Nursing, University of Massachusetts Amherst, Amherst, MA 01003, USA; 3 William F. Connell School of Nursing, Boston College, Chestnut Hill, MA 02467, USA; andrew.dwyer@bc.edu; 4 P50 Massachusetts General Hospital, Harvard Center for Reproductive Medicine, Boston, MA 02114, USA

**Keywords:** genetics, genomics, health disparities, intersectionality, identities, *BRCA* testing

## Abstract

**Simple Summary:**

Genetic testing for *BRCA1/2* is recommended for individuals at high risk of hereditary breast and ovarian cancer, yet racial and ethnic disparities persist. Diverse and mutually influencing factors contribute to *BRCA* health disparities, including socially constructed identities (e.g., gender, race, and ethnicity), sociodemographic factors (e.g., income and education), and societal factors (discrimination). Such factors can be additive and multiplicative. Viewing *BRCA* health disparities through an intersectional lens may provide a more nuanced, comprehensive understanding of lived experience by considering individuals’ unique, intersecting identities. A deeper understanding of the individual can inform more person-centered approaches to genomic healthcare and help bridge *BRCA* health disparities.

**Abstract:**

Significant health disparities exist in relation to pathogenic variants in *BRCA1/2*. This study aimed to better understand the barriers and facilitators to *BRCA1/2* genetic testing and intrafamilial communication of risk in racially and ethnically diverse individuals. We conducted qualitative interviews with non-Hispanic White (*n* = 11) and Black, Indigenous, People of Color (BIPOC) individuals (*n* = 14) who underwent testing for pathogenic *BRCA1/2* variants. We employed template analysis, case study analysis, and comparative case study analysis to examine healthcare experiences related to genetic testing as well as intrafamilial communication of risk. Applying an intersectional lens, we sought to inform more person-centered approaches to precision healthcare and help dismantle disparities in genomic healthcare. Template analysis revealed salient factors at the individual (psychosocial well-being), interpersonal/familial, and healthcare system levels. A two-part case study analysis provided insights into how race/ethnicity, cultural norms, and socioeconomic status interact with systemic and structural inequities to compound disparities. These findings underscore the need for person-centered, tailored, and culturally sensitive approaches to understanding and addressing the complexities surrounding testing and the communication of *BRCA* risk. Applying an intersectional lens can inform more person-centered approaches to precision healthcare and may help to surmount existing disparities.

## 1. Introduction

Pathogenic variants in *BRCA* 1/2 confer high risks of hereditary breast and ovarian cancer (HBOC) and warrant risk reduction strategies and personalized surveillance for hereditary cancers [1]. Guidelines recommend testing for *BRCA* variants in those at increased risk for cancer as findings are likely to impact risk management and the treatment of tested individuals and their family members [2,3]. Intrafamilial communication of *BRCA*-related risk enables the testing of blood relatives (i.e., cascade carrier screening) and early interventions to reduce cancer risk and mortality [3].

Notably, non-Hispanic Black (NHB) and Hispanic individuals suffer increased mortality from breast cancer at every stage compared to their non-Hispanic white (NHW) counterparts [4,5]. Further, rates of *BRCA* variants are higher among Black females compared to NHW [6,7] and individuals not of Ashkenazi Jewish ancestry [8]. Studies on genetic testing for hereditary cancers demonstrate that genetic testing is underutilized among racially minoritized NHB and Hispanic populations compared to NHW people [5,6,9,10,11,12]. A range of social identity factors, including race, age, cultural beliefs, and socioeconomic status (SES), contribute to genetic testing disparities and intrafamilial communication of *BRCA* risk [13,14]. Such factors may intersect, resulting in compounding effects for historically minoritized racial and ethnic groups.

Intersectionality is a theoretical framework that provides a layered understanding of how aspects of a person’s identity (e.g., age, race, ethnicity, gender, class, and ability) intersect to influence an individual’s privilege and experiences of discrimination [15]. Intersectionality posits that socially constructed identities (e.g., gender and ethnicity), social categories (e.g., income and education), and social processes (e.g., stigma and discrimination) exist together and contribute to health inequities [16]. Unlike approaches that consider aspects of identity in isolation, intersectionality highlights the multiplicative impacts of one’s identity on lived experience. An intersectional lens recognizes that racial and ethnic groups are not uniform, monolithic entities but are composed of individuals with unique lived experiences. For example, the experiences of younger people may differ significantly from those of older adults in the same community. Likewise, economic and educational differences can uniquely shape the experiences of individuals within the same racial or ethnic group. Considering an individual’s unique identities and intersections provides a more nuanced, comprehensive understanding of lived experience and avoids ‘essentializing’ an entire group. Using an intersectional lens as a clinical and research framework may facilitate a multi-dimensional, individualized, and holistic approach to caring for people affected by cancer [17]. Importantly, such individualized perspectives align with the tenets of precision healthcare that emphasize approaches that are targeted and tailored to the individuals’ needs [18].

Adopting an intersectional lens may facilitate person-centered approaches to precision healthcare that are equitable and bridge existing disparities. To date, few research studies have used an intersectional approach to examine disparities in genomic healthcare. This study aims to gain a deeper understanding of barriers and facilitators to *BRCA* genetic testing and intrafamilial communication of *BRCA* risk in diverse individuals. We apply an intersectional lens to analyze qualitative interviews with individuals who underwent *BRCA* testing to inform more person-centered approaches to precision healthcare and help dismantle disparities in genomic healthcare.

## 2. Materials and Methods

This work is the qualitative component of a sequential, mixed-methods (i.e., quantitative and qualitative approaches) study examining racial and ethnic disparities in genomic healthcare utilization, patient activation, and intrafamilial communication risk among females tested for *BRCA* variants [19]. This study was approved by the Boston College Institutional Review Board (IRB# 16.109.01). Opt-in online informed consent was obtained from all participants prior to study participation.

### 2.1. Sample and Data Collection

The sample and data collection for the mixed-method study have been previously reported [19]. Briefly, the sample included English-speaking adults (18+ yrs.) who underwent genetic testing for *BRCA1/BRCA2*. We employed community-engaged outreach in collaboration with trusted patient support organizations. Following opt-in informed consent, participants completed a quantitative online survey to record information on sociodemographics, medical history, genetic testing, family communication, and several validated instruments. We invited a subset of participants (*n* = 25) to be interviewed (between February 2022 and May 2023).

In-depth qualitative interviews (45–90 min.) were conducted to gather detailed narratives on personal experiences with the genetic testing process, emotional and coping responses, intrafamilial communication of risk, and healthcare services. Open-ended prompts guiding semi-structured interviews are depicted in Table 1. Interviews were transcribed verbatim and combined with field notes for qualitative analyses.

### 2.2. Data Analysis

**Figure 1:**
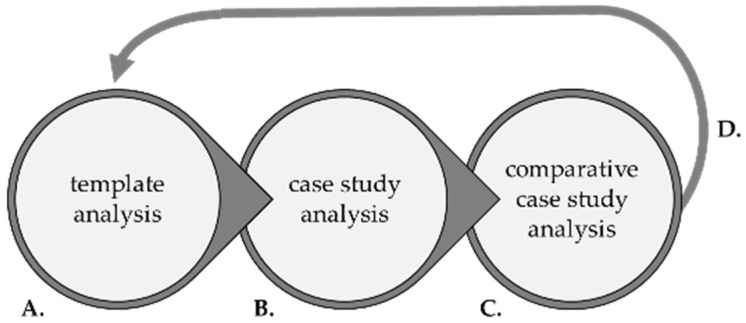
Approach to qualitative analysis. (**A**) Template analysis provides a broad, inclusive overview of themes from the existing literature yet lacks social context. (**B**) Case study analysis provides in-depth insights (including social context) into lived experiences described in each case. (**C**) Comparative case study analysis compares/contrasts cases to identify similarities, differences, and patterns across cases. (**D**) Insights from comparative case study analysis contextualize and illuminate broad themes from template analysis (literature).

Multiple qualitative analytic approaches (template analysis, case study analysis, and comparative case study analysis) were employed to analyze interview data (Figure 1). Each analytic approach offers a different perspective, and, when combined, the integrated analysis contributes to a more holistic, nuanced understanding of complex phenomena.

The intersectionality theoretical framework guided our approach to qualitative analyses to elucidate both the heterogeneity of effects and potential causal processes underlying health inequities [16]. The framework provides a critical, unifying interpretive and analytical lens that reframes how social and health inequities are examined, analyzed, and addressed.

First, transcripts of qualitative interviews were analyzed using template analysis [20,21]. Template analysis consists of both a deductive coding of interview transcripts to identify dimensions, patterns, and variations of a priori ‘template’ themes derived from the existing literature and an inductive coding that allows for emergent themes to arise based on the lived experiences of participants. In this study, template analysis focused on the barriers/facilitators of genetic testing for *BRCA* and the intrafamilial communication of hereditary cancer risk. Investigators (SH-B and HS) independently coded interview transcripts to support analytic rigor and validity. Coding disagreements were resolved by discussion with other study investigators (MS and AAD). Multiple investigator perspectives support comprehensive and robust interpretation. Template analysis facilitates viewing themes across the entire dataset, reducing the need for extensive coding, and making it easier to analyze large qualitative datasets. A limitation of this approach is that coded content may lack social context and connection to real-life experiences.

Following template analyses to identify barriers/facilitators at different levels (individual, interpersonal, and healthcare system), we employed a two-part case study approach (i.e., case study analysis and comparative case study analysis) [22,23]. Complementary analytic approaches were employed to capture the spectrum of lived experiences and to better understand the role and interaction of various barriers/facilitators individuals encounter in their *BRCA* testing journey. An in-depth analysis of a single case study aims to understand an individual’s lived experience and provide deep insights into issues not readily identified via other methods. For case study analyses, we purposefully selected varied participants based on ethnicity, age, and socioeconomic status (i.e., income). Cases were selected to showcase the interplay and complexities of challenges and support across different aspects of participants’ lives and healthcare journeys (e.g., psychosocial factors, family dynamics, and healthcare access). Unlike template coding, which may exclude contextual information, case study analysis provides an in-depth examination of participants’ lived experiences. In the present study, we employed case study analysis to illuminate the ways multiple factors intersect and impact overall *BRCA*-related experiences. However, it is worth noting that case study findings are not generalizable, as findings from a limited number of case studies may lead to stereotyping experiences and outcomes.

Accordingly, we subsequently conducted a comparative case study analysis to explore similarities and differences across cases. Comparative analysis involves comparing/contrasting different cases to identify similarities and differences to uncover patterns, distinct shared themes, and underlying structural factors. We analyzed similarities by looking for common, shared patterns, themes, and outcomes across individual cases. We analyzed differences by examining variations in context, experiences, and outcomes. Specific examples are highlighted and contextualized using direct quotes to support findings and provide examples of how intersectionality plays out across different cases.

## 3. Results

The sociodemographic and clinical characteristics of the 25 participants are shown in Table 2. The sample included 11 non-Hispanic White and 14 Black, Indigenous, People of Color (BIPOC) individuals who underwent BRCA testing.

### 3.1. Template Analysis Results

Qualitative template analyses revealed barriers centered on genetic testing decisions, communicating results with family, advocating for services, and adapting to learning one’s BRCA status. Barriers and facilitators were identified at the (1) individual, (2) interpersonal/familial, and (3) healthcare system levels. Individual level themes were related to identity, coping strategies, emotional responses, and psychosocial well-being during/after genetic testing. Interpersonal/family level themes included factors related to family characteristics and dynamics (e.g., norms) affecting an individual’s testing decision and subsequent intrafamilial communication of BRCA-related cancer risk. Healthcare level themes included interactions with providers and the healthcare system (e.g., access, trust) affecting testing decisions, risk communication with family/providers, and overall satisfaction with healthcare experiences. Facilitators increased the likelihood of having genetic testing, communicating risk within the family and to healthcare providers, and reflected generally positive healthcare experiences. Supplementary Table S1 depicts themes (barriers/promoters) and representative quotes for each barrier and promoter at different levels (individual, interpersonal/family, and healthcare system).

### 3.2. Case Study Analysis: Selected Individual Cases

**Figure 2:**
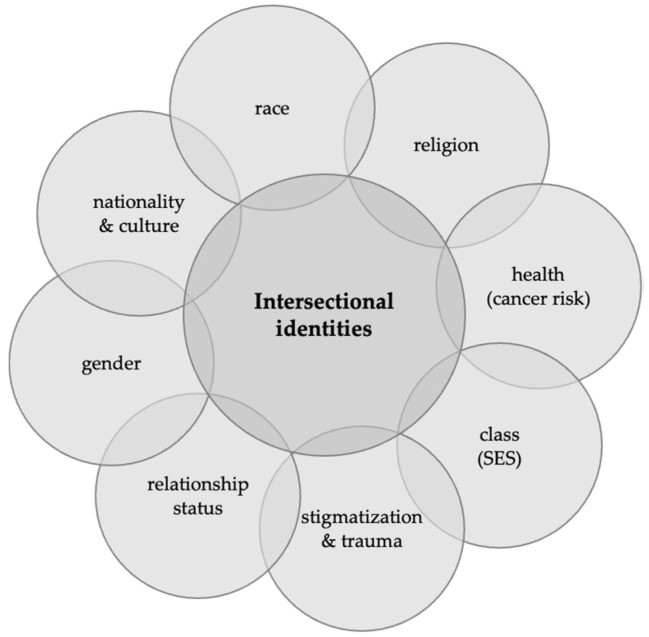
Schematic depicting intersectional identities. An intersectional lens affords insight into individual-level responses (e.g., emotional and coping responses), interpersonal-level behaviors (e.g., intrafamilial communication of risk), and interactions with healthcare providers and systems (i.e., trust/distrust, access). Notably, each element of one’s identity may contribute protective (e.g., strong social support, financial sources, and optimism) or insidious effects (e.g., low patient activation, fatalism, and avoidant coping).

We used a case study analysis to mitigate the risk of oversimplifying patient experiences in template analysis. Examining individual cases from varied ethnic/racial backgrounds, we applied an intersectional lens to varied interconnected factors affecting lived experiences. Case study analysis highlights how individuals simultaneously navigated multiple barriers and facilitators and provides insight into how their identities shape overall experiences in additive and multiplicative ways (Figure 2).

#### 3.2.1. Case Study 1 (ID#001)

Participant #001 is a 68 year-old Mexican-American woman with an annual income < USD 25,000. She had been diagnosed with both breast and ovarian cancer and was found to be BRCA+ on genetic testing. While her limited financial means and insurance coverage restricted her access to high-quality care, her humor facilitated her active coping response. Several intersecting aspects of her identity (i.e., older age, Mexican-American background, lower socioeconomic status, and cancer diagnoses) shaped her BRCA experience. Despite barriers, she described proactive, active coping responses, including actively seeking out genetic testing and using the internet to find information on cancer and increase her health literacy. However, she expressed a desire for more guidance, emotional support, time, and explanations from healthcare providers—pointing to health system barriers in her cancer journey.

At the individual level (i.e., psychosocial well-being), she expressed feeling disempowered—noting how her finances, poor insurance, and isolation often limited her options and determined her healthcare decisions. Intersecting elements underscore the intertwined, adverse effects that contributed to her feeling marginalized; “If I was someone important, or if I was the wife of such and such and so and so… They would be checking my fingernails”. She distanced herself from a cancer support group because she felt the sadness expressed by group members exacerbated the significant emotional strain of her dual cancer diagnosis and BRCA+ test results.

At the interpersonal/familial level, intersections between age, ethnicity, and family culture shaped norms, making it difficult to communicate BRCA risk to family members. The lack of intrafamilial communication of risk is emblematic of privacy norms surrounding health matters within certain cultures. She shared that critical health history had not been shared in her family—contributing to her lack of understanding of hereditary cancer risk for herself and her daughter (who died from ovarian cancer at a young age). However, her identity as a prominent elder contributed to her sense of empowerment within the extended family structure.

At the healthcare system level, she described the compounding of obstacles of ethnicity and socioeconomic status (SES). Her Mexican heritage and low SES both contributed to her feeling marginalized in healthcare interactions. She lamented her lack of adequate health insurance coverage. She felt that crucial information pointing to hereditary cancer risk was in her medical record “way back” and was not discussed because of combined biases against older, lower-income, Mexican-American women.

#### 3.2.2. Case Study 2 (ID#003)

Participant #003 is a 28-year-old BRCA+ woman with a mixed racial/ethnic background comprising White, Black, and Hispanic heritage. She reported her annual income as USD 25,000–50,000. She described how her age, heritage, gender, and SES affected her psychosocial well-being, family dynamics, and healthcare journey alike.

At the individual level, her psychosocial well-being was shaped by her young age, gender, race/ethnicity, and SES. In terms of age, she described pressures around decisions (i.e., risk-reducing surgery) that directly impact her body image, fertility decisions, and sense of self, noting, “Once you remove that [breasts], you accept… you’re accepting that you’re not going to be the same… I just wish I could have kids the way… my whole life has not been easy”.

At the interpersonal/family level, intersecting identities of young age, gender, cultural norms, and low SES affected the intrafamilial communication of risk; “It’s [BRCA] difficult because we simply do not talk about it”. In this case, family norms involved not discussing health issues with younger family members. Notably, her parents were aware of BRCA in the family yet grappled with guilt for passing the risk on. Her father’s guilt was compounded by gender scripts wherein he saw himself as “the family protector” for his daughter, which posed barriers to open communication. Moreover, the family’s low SES contributes to barriers to accessing medical care and resources—an aspect that appeared to magnify the Father’s feelings of guilt (i.e., not providing for the family) and blocked communication.

At the healthcare system level, a lack of financial resources compounded the challenges she faced from racial/ethnic biases relating to genetic risk; “I felt like my genetic counselor did not think I would test positive because I am not Ashkenazi and because of my ethnic factors”. In relation to her age and gender, she explained feeling that her surgeon’s treatment recommendation was distressing and did not consider her identity as a young woman, “When I consulted the surgeon, she strongly recommended that I…not keep my nipples”. Such interactions left her feeling “excluded” from healthcare services.

#### 3.2.3. Case Study 3 (ID#009)

Participant #009 is a 30-year-old African American woman with an annual income of USD 101,000–125,000. She had genetic testing at the age of 25 based on her family history of breast cancer and was found to be BRCA+. From an intersectional perspective, her age, race, gender, family medical history, and SES had a combined impact on her BRCA journey.

At the individual level, cultural and community factors were strong influences. She expressed a sense of fatalism in relation to being a Black person of faith; “Most of the time, things are planned out for us”. Moreover, she expressed deep concerns about how mastectomy would impact her body image; “I have never envisioned myself without a breast”. Her statement highlights gendered pressures associated with femininity and beauty that may be amplified by cultural norms in the Black community. Thus, while her high income provides her with resources (e.g., counseling reconstructive surgeries), SES does not inoculate her against psychological distress related to societal/cultural pressures and expectations.

At the interpersonal/family level, the intersection of her age, race, gender, family medical history, and SES complicated her genetic testing experience and communication of genetic risk. She described how identifying as Black carries a cultural history that shaped her healthcare decisions. She explained that women were central in healthcare decisions in her family and culture. Additionally, her mother’s experience as a BRCA+ breast cancer survivor was a strong influence, “My mom got cancer when she was quite older… Then, we went ahead with those [genetic] tests”. She shared the challenges of navigating expectations for having children, describing a sense of urgency because she is BRCA+ and faces treatment decisions that will impact fertility. Despite her considerable financial resources, she described struggling with the emotional and psychological burden of being BRCA+, as well as treatment decisions and sequelae of risk-reducing interventions.

At the healthcare system level, intersecting aspects of her identity impacted how she navigated a complex healthcare ecosystem. She described how she actively sought out cancer information and was learning about how genes function. One could attribute such active coping responses to her SES, which likely provided her with additional resources. Further, her proactive approach suggests resilience—an important facet of dealing with potential biases that people of color may encounter in the healthcare system.

#### 3.2.4. Case Study 4 (ID#011)

Participant #011 is 36 year-old woman who identifies as Black. She holds a master’s degree in public health and has an upper middle class income (USD 76,00–100,000). Her father is a prostate cancer survivor, and her mother tested *BRCA2*+ and died from breast cancer at the age of 52. She described witnessing a distressing encounter between her mother and a physician who seemed dismissive. She was deeply impacted by the experience that she attributed to racial bias. At age 22, she tested positive for BRCA2. She had not been diagnosed with cancer, yet felt challenged to come to terms with her BRCA+ status at such a young age.

At the individual level, her empowered ability to advocate for herself helped her navigate challenges associated with being BRCA+. She perceived that her healthcare providers held implicit biases, and she described feeling slighted at the time of testing because of her young age. However, she has a master’s degree in public health and high health literacy that contributed to her feeling empowered to address structural inequities in the healthcare system and her care. In this case, her educational attainment, career, and SES served as buffering, protective factors providing her with a certain degree of privilege.

At the interpersonal/family level, the family cancer history, norms, and values shaped her BRCA experience. Both of her parents were educators who actively sought information on BRCA and treatment options for her mother. The family valued knowledge and served as a role model for information seeking and an active coping response. The advantages conferred by educational attainment and SES enabled a deeper understanding of hereditary cancer risk. Families lacking such resources and advantages may lack the knowledge and capacity to research options.

At the healthcare system level, the convergence of her young age, gender, role as a mother, and racial identity touch on various forms of oppression; “As I have navigated through the healthcare system over the two years while becoming an adult and having two children, I am acutely aware that I am a Black woman”. While her SES may provide privilege and access to healthcare, she described feeling stigmatized and treated dismissively because she was a young Black mother. She shared experiences of racism, not from her healthcare providers but in navigating the healthcare system. In this case, her privilege (SES) did not fully shield her from racism and sexism.

#### 3.2.5. Case Study 5 (ID#025)

Participant #025 is a 36-year-old Mexican woman who joined the U.S. Navy to have access to healthcare and a college education. Both her mother and maternal aunt died from breast cancer, yet the presence of BRCA within the family remained obscured. Her story highlights the interplay between personal experiences, family history, and systemic influences on her BRCA journey.

At the individual level, the convergence of age, cultural beliefs/norms, and SES shaped her coping response and resilience. She noted that growing up poor, she was in a constant state of vulnerability and felt responsible for her and her younger sister’s survival. She described how the family history of breast cancer also shaped a fatalistic perspective. She felt that because she bore a close physical resemblance to her mother, her eventual BRCA1+ test result was “inevitable”.

At the interpersonal/family level, the interconnectedness of ethnicity, cultural norms, and SES shaped family communication. She shared how language barriers and being a Mexican immigrant influenced her healthcare decisions. She also shared difficulties in communicating *BRCA* risk to Spanish-speaking family members: “I had to request information in Spanish to explain this genetic risk to my family in Mexico”. However, shared cultural values also fostered close familial bonds that were a source of support: “My sisters and their families provide support in making [medical] decisions”.

At the healthcare system level, her career, education, and SES provided a range of advantages as well as obstacles. Being in the U.S. Navy, she had access to healthcare through the Veteran’s Administration (V.A.), yet she described limitations; “I mean, they’re only authorized to do specific things”. She described feeling more equipped to navigate the healthcare system than other family members because of her language/literacy abilities and college education. She described how such advantages helped her feel empowered to seek surgical care beyond the V.A. healthcare system.

### 3.3. Qualitative Comparative Analysis of the Case Studies

We employed comparative case study analysis to identify patterns and shared themes (i.e., barriers/facilitators) across different participants and levels (i.e., individual, interpersonal/family, and healthcare system). Although identified factors were not necessarily exclusive to a single level (i.e., intersectionality), barriers and facilitators were reported across levels. A detailed summary of comparative case studies is provided in Table 3.

#### 3.3.1. Mitigating Barriers at the Individual Level

Cultural beliefs (participants #001 and #009), fatalistic beliefs (#009 and #025), and the emotional burden of ethnic/racial identity (#003 and #011) posed barriers for participants in their respective BRCA journeys. Barriers affected health decisions and preventive care attitudes and behaviors (#001 and #009)—including risk-reducing surgery decisions. Greater health literacy, resilience, and active coping responses at the individual level were protective factors that mitigated barriers across different ethnic/racial identities (#001, #003, and #009) in the healthcare system. Health literacy enabled participants to navigate the complexities of genetic risk, medical decisions, and healthcare services. Regardless of racial/ethnic background, resilience and active coping mechanisms (i.e., information seeking, being proactive, self-advocacy, and humor) emerged as significant facilitators of well-being (Table 3).

#### 3.3.2. Mitigating Barriers at the Interpersonal/Family Level

Family norms inhibiting open communication, lack of support from family members, limited awareness of BRCA risk, and negative attitudes towards genetic testing all impacted participants’ psychological well-being at the individual level, as well as intrafamilial communication of BRCA risk. Cultural factors play a significant role in conveying genetic risk within families. Specifically, cultural views that stigmatize genetic testing/conditions (#001, #003, and #011) and/or limit family communication/support (#001 and #003) impede sharing BRCA risk. A comparative analysis showed a similar lack of awareness/knowledge regarding genetic risk across communities, suggesting structural gaps in health education (#001, #003, #009, and #025). Resistance to testing was evident among family members identifying as Hispanic/Latino and Black, which appear to be influenced by cultural beliefs, SES, and/or distrust in healthcare (#001, #003, #009, and #025). In contrast, open and supportive communication with immediate family members (#003, #011, #009, and #025) and proactive risk management (#001 and #009) helped individuals to effectively communicate BRCA risk (and risk reduction) to family members. Across cases, supportive family members appear to be crucial for high-quality decisions (i.e., informed and aligned with values and preferences) for genetic testing and risk management (Table 3).

#### 3.3.3. Mitigating Barriers at the Healthcare System Level

A number of shared experiences contribute to difficulty in navigating the healthcare system and making health decisions. Common experiences included perceived systemic bias, implicit bias/racial discrimination, lack of culturally sensitive care, dissatisfaction with genetic counseling, and socioeconomic constraints. Participants (#009 and #011) expressed the need for enhanced training of staff and providers on culturally empowered approaches to care. Black participants (#011 and #018) described experiences of systemic discrimination manifesting as implicit biases and micro-aggressions. However, receiving person-centered and culturally sensitive care contributed to more positively regarded experiences and greater satisfaction with the care supporting intrafamilial risk communication (#011). Regardless of racial/ethnic identity, financial aspects (i.e., low SES and limited insurance coverage/access to healthcare) were cited as key barriers (#001, #003, and #025). Conversely, higher SES (#005) had a buffering effect on intersecting barriers by facilitating healthcare access (e.g., private comprehensive insurance). Similarly, greater educational attainment and higher perceived health literacy enabled participants to effectively navigate care (#001, #003, #009, and #011). Self-advocacy appeared to correspond with the ability to make complex healthcare decisions and communicate hereditary cancer risk to blood relatives (Table 3).

## 4. Discussion

We explored the experiences of diverse individuals who underwent genetic testing for *BRCA* 1/2 gene variants. Applying an intersectional lens, we identified barriers and facilitators related to genetic testing decisions, intrafamilial communication of *BRCA* genetic risk, and experiences of both privilege and discrimination in healthcare. Participant narratives reveal how multiplicative impacts of intersecting identities affect individual attitudes, beliefs, emotional and coping responses, and health behaviors. These multiplicative impacts are also present at the interpersonal/familial level, affecting intrafamilial communication of risk and interactions with healthcare providers. Intersectionality also influenced how participants navigated the continuum of care in a complex healthcare ecosystem—from genetic testing to risk-reducing interventions, to cancer treatment, to cancer survivorship.

Prior studies show that the genetic counseling process, genetic testing, and revelation of *BRCA*+ status elicit a range of negative emotional responses (i.e., decisional conflict, shock, and distress) [24,25,26]. Notably, avoidant coping strategies (i.e., denial and concealment) in response to learning one’s *BRCA+* status are associated with poorer health status, decreased physical function, and diminished health-related quality of life [27]. In contrast, active coping responses (i.e., seeking information and support, and self-advocacy) are associated with satisfaction with health decisions, psychological adaptation, and improved well-being [28]. Prior quantitative work indicates that individuals with higher satisfaction with genetic testing decisions, lower fatalism, and higher resilience are more likely to have greater knowledge, skill, and confidence in managing a health condition (i.e., patient activation) [19]. In the present study, complementary qualitative analytic approaches reveal how the intersecting aspects of an individual’s identity (e.g., age, race, ethnicity, gender, and culture) act in a multiplicative manner, conferring both protective, buffering aspects (promoting well-being and risk communication) and/or negative consequences on coping/emotional responses that limit intrafamilial communication of *BRCA* risk.

**Figure 3:**
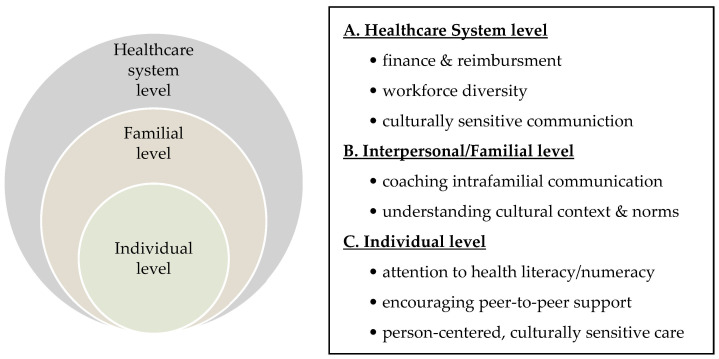
Multilevel goals derived from qualitative findings. (**A**) Health system interventions may focus on reducing financial barriers to genetic counseling and testing, building a diverse and skilled healthcare workforce, and training them in culturally sensitive communication skills. (**B**) Interpersonal/familial level interventions may center on healthcare providers modeling understanding cultural context and norms, shared decision-making, and offering coaching to build confidence for intrafamilial communication of risk. (**C**) Individual level interventions should be mindful of health literacy and numeracy skills, encouraging peer-to-peer support, and tailoring an approach to intersectionality to support psychosocial well-being.

Applying an intersectional lens identified targets for person-centered interventions as a part of precision healthcare (Figure 3). Person-centered care is tailored and coordinated, it recognizes individuals as equal partners, and respects their potential and existing capabilities to manage and improve their health and well-being [29]. Thus, person-centered precision healthcare extends beyond targeted treatments (based on molecular and genetic factors) and recognizes diverse sociocultural influences on health [30]. Further, an intersectional perspective can promote precision healthcare that is responsive and equitable and helps to reduce disparities in genomic healthcare [18].

Intrafamilial communication of risk is critical for reaping the full benefit of genetic testing. Genetic counseling and testing for relatives who may also be at increased cancer risk should be discussed with individuals who test positive for *BRCA* 1/2 variants [3]. The U.S. Preventive Services Task Force (USPSTF) recommends that individuals with a family history of *BRCA1/2* gene variants be assessed with a validated risk assessment tool to inform cascade screening [2]. Cascade screening relies on individuals testing positive for *BRCA* variants to communicate their risk to blood relatives who may be at risk. Findings from the present study underscore how culture (i.e., presence/absence of supportive family members and family norms) shapes the intrafamilial communication of risk. Supportive family and favorable family attitudes and beliefs towards genetic testing are influential as individuals often feel a sense of solidarity with family members [24] that facilitates the disclosure of test results [31]. Participants in this study described a mix of familial and cultural obstacles, including resistance from family members, concern about family reactions, cultural attitudes/beliefs, as well as limited awareness, information, and communication of family *BRCA* risk and the importance of sharing test results. Cultural factors, including different traditions, norms, and taboos merit consideration. Participants from racially and ethnically minoritized groups emphasized the significance and importance of knowing their family history as a key factor supporting the intrafamilial communication of risk. These data suggest the need for culturally empowered approaches that consider culture as a mediating force for genetic testing and the intrafamilial communication of risk.

At the healthcare system level, qualitative findings highlight the role that providers and healthcare systems play in shaping patient experiences, satisfaction, and the ability to access timely care. Guidelines from the National Comprehensive Cancer Network (NCCN) provide evidence-based recommendations for healthcare professionals regarding genetic testing criteria, follow-up, and the management of individuals who would benefit from genetic testing [3]. Personal history of cancer (at any age) and specific features (i.e., one or more blood relatives with breast cancer diagnosed ≤50 yrs.) are clinical indicators warranting genetic testing [3]. The NCCN guidelines also recommend genetic testing for individuals without a personal cancer history who have a family history of cancer in first- or second-degree blood relatives [3]. Mittendorf et al. [32] describe a range of clinician and healthcare system factors that contribute to health inequity. The review notes that health insurance coverage, access to care, socioeconomic status, educational attainment, and clinician–patient communication are key drivers of inequity and disparities in genetic healthcare. McCarthy et al. [11] found that Black women were less likely than White individuals to receive recommendations for *BRCA* 1/2 testing. Similarly, Black women with a personal history of cancer are less likely to undergo genetic testing [6]. To bridge documented disparities in *BRCA* testing rates, efforts should be directed toward removing financial and administrative obstacles hindering access to genetic testing services and culturally sensitive approaches emphasizing the benefits of genetic testing [33]. Notably, we identified elements of systemic racism in patient narratives. Thus, renewed efforts should address distrust and enhance cross-cultural communication across the continuum of care (i.e., referral, genetic counseling, genetic testing, and result disclosure) [34].

Racial and ethnic groups are not uniform, and individuals have unique lived experiences and varied intersectional identities within a larger cultural group [35]. For example, the experiences of younger individuals may differ significantly from those of elders within the same cultural community. Similarly, gender, economic, and educational differences can uniquely shape experiences among individuals from the same racial or ethnic demographic. In this study, the experiences of participants highlighted targets for developing more person-centered, culturally empowered approaches to care. Such approaches consider multiple aspects of identity, including race/ethnicity, socioeconomic status, and cultural community norms. Compounding oppressive factors work at multiple levels and likely require multilevel interventions to mitigate disparities and more fully support the health and well-being of diverse individuals.

## 5. Limitations

Our research approach, which combines a theoretical framework and dual analytical methods, allowed us to thoroughly examine the lived experiences of individuals. There are several limitations to this work. First, compared to quantitative studies, the sample size for qualitative investigations is smaller. It merits noting that qualitative research findings should be considered transferable rather than generalizable. However, our primary focus was on gaining in-depth insights through an intersectional lens rather than aiming for broad generalizability. Second, we relied on self-reported data, which may introduce bias related to recall or social desirability. We implemented measures to establish rapport and create an open, transparent environment to minimize the risk of these potential biases. Additionally, it is important to acknowledge that the experiences of individuals with *BRCA* mutations can vary based on geography and the local context of their respective healthcare systems, cultural attitudes towards genetic testing, and community support for hereditary cancer. These limitations merit consideration when interpreting the study findings.

## 6. Conclusions

Findings from this study suggest how the multiplicative effects of intersectional identities may compound *BRCA* disparities. Individual identities impact BRCA genetic testing decisions, family risk communication, and overall healthcare experiences. Efforts focusing on the education and awareness of genetic risk and testing may not be sufficient. Findings underscore the need for tailored, culturally sensitive approaches to reap the full benefit of cascade screening. Applying an intersectional lens can inform more person-centered approaches to precision healthcare and may help to surmount existing disparities.

## Figures and Tables

**Table 1:** Interview guide.

Open-ended interview prompts
Can you talk a little bit about your genetic testing history as you remember it? Have you been diagnosed with any cancer? How did you decide to get tested? How would you describe your testing process in the healthcare system? Have you seen a genetic counselor? How was your experience with healthcare professionals? Have you felt anything related to your race/ethnicity, age, gender, or insurance affecting your experiences during the testing process? How would you describe your experience with having your test results? How did you feel about your test results physically, emotionally, and psychologically? Have you had any treatments such as surgery related to your BRCA status? How did you respond to it physically, emotionally, or psychologically? Have you become involved with any BRCA or cancer activist/support groups? What has your experience in these groups been like? Have you communicated your test results with your family? If so, how did you decide who to communicate with? How would you describe this communication process? How do you feel during this process with reactions from family members? Can you talk a little bit about how this testing process and test results affect your family relationship, if any? Is there any aspect of your personal journey we have not discussed? Is there anything you want to share with us affecting the overall experience during testing and after your test results?

**Table 2:** Socio-demographic and clinical characteristics of participants (*n* = 25).

Characteristics	*n* (%)
**Age (years)**	
min.–max.	25–68
**Race/ethnicity**	
Non-Hispanic white	11 (44%)
African American/Black	7 (28%)
Hispanic/Latino	1 (4%)
Asian or Asian American	3 (12%)
Mixed	1 (4%)
missing	2 (8%)
**Gender (current)**	
female (*cis*)	20 (80%)
male	2 (8%)
missing	3 (12%)
**Marital status**	
married/in a relationship	15 (60%)
single	5 (20%)
separated/widowed	2 (8%)
missing	3 (12)
**Education**	
some college/Associate’s degree	3 (12%)
college or advanced degree	19 (76%)
missing	3 (12%)
**Household income (annual)**	
<$ 75,000/yr.	9 (36%)
USD 75,000–125,000/yr.	8 (32%)
>USD 125,000/yr.	6 (24%)
missing	2(8%)
**Pathogenic *BRCA* variant**	
yes	20 (80%)
no	2 (8%)
missing	3 (12%)
**Personal history of cancer**	
yes	7 (28%)
no	15 (60%)
missing	3 (12%)

**Table 3:** Barriers/facilitators at the individual, interpersonal/family, and healthcare system levels across cases.

*Case*	*Barriers*	*Facilitators*
	Individual Level	
*#001*	-cultural beliefs *(intersecting with healthcare level)* -fatalism -emotional burden (*BRCA*+ status)	-resilience *(intersecting with healthcare level)* -active coping strategies
*#003*	-emotional burden (unspoken ‘heaviness’)	-resilience *(intersecting with healthcare level)* -active coping strategies
*#009*	-cultural beliefs (genetic testing) -fatalism	-resilience -active coping strategies
*#011*	-emotional burden (*BRCA*+ status)	-resilience *(intersecting with healthcare level)* -active coping strategies -self-advocacy (*intersecting with healthcare level*)
*#025*	-fatalism -frustration with uncertainty	-resilience *(intersecting with healthcare level)* -active coping strategies -self-advocacy (*intersecting with healthcare level*)
	Interpersonal/familial Level	
*#001*	-language barriers to risk communication *(intersecting with healthcare level)* -lack of awareness of genetic risk -family resistance to genetic testing	-proactive risk management
*#003*	-barriers to communicating genetic risk -lack of awareness of genetic risk -family resistance to genetic testing	-cancer risk awareness *(intersecting with individual level)* -family norms of open communication
*#009*	-family resistance to genetic testing -cultural beliefs *(intersecting with* *individual & healthcare levels)*	-proactive risk management -awareness of *BRCA* risk *(intersecting with individual level)* -open communication
*#011*	-barrier to communication of genetic risk -complexity of genetic information *(intersecting with individual level)*	-open, family communication -supportive family members
*#025*	-initial lack of awareness about genetic risk *(intersecting with individual level)* -familial resistance to genetic *t* testing	-awareness of *BRCA* risk *(intersecting with individual level)* -open family communication of risk
	Healthcare system Level	
*#001*	-dissatisfaction with genetic counseling -lack of culturally sensitive care -financial constraints	-high health literacy *(intersecting with individual level)* -resilience *(intersecting with individual level)* -active coping strategies
*#003*	-dissatisfaction with genetic counseling -socioeconomic constraints -systemic bias	-high health literacy *(intersecting with individual level)* -resilience *(intersecting with individual level)* -active coping strategies
*#009*	-systemic bias	-high health literacy *(intersecting with individual level)* -proactive risk management
*#011*	-lack of rapport with provider -systemic racism	-culturally sensitive care *(intersecting with familial level)*
*#025*	-socioeconomic constraints *(intersecting with individual level)* -difficulty obtaining genetic testing referral	-high health literacy *(intersecting with individual level)* -quality care

#001: 68 y.o. Mexican female (<$ 25,000/yr); *BRCA*+, no personal history of cancer; #003: 28 y.o. mixed race/ethnicity female ($ 25,000–50,000); *BRCA*+, no personal history of cancer; #009: 30 y.o. Black female ($ 101,000–125,000); *BRCA*+, positive personal history of cancer; #011 35 y.o. black female ($ 76,000–100,000), *BRCA*+, no personal history of cancer; #025 36 y.o. Hispanic/Latino female (“low” income), *BRCA*+, no personal history of cancer.

## Data Availability

De-identified data will be made readily available to qualified individuals within the scientific community upon request for research purposes.

## Supplementary Materials

The following supporting information can be downloaded at https://www.mdpi.com/article/10.3390/cancers16091766/s1, Table S1: Themes and correspondent category of template analyses of participants’ experiences.

## References

[1] Kuchenbaecker K.B., Hopper J.L., Barnes D.R., Phillips K.A., Mooij T.M., Roos-Blom M.J., Jervis S., van Leeuwen F.E., Milne R.L., Andrieu N. (2017). Risks of Breast, Ovarian, and Contralateral Breast Cancer for BRCA1 and BRCA2 Mutation Carriers. JAMA.

[2] Owens D.K., Davidson K.W., Krist A.H., Barry M.J., Cabana M., Caughey A.B., Doubeni C.A., Epling J.W., Kubik M., US Preventive Services Task Force (2019). Risk Assessment, Genetic Counseling, and Genetic Testing for BRCA-Related Cancer: US Preventive Services Task Force Recommendation. JAMA.

[3] The National Comprehensive Cancer Network® (NCCN®), Clinical Practice Guidelines in Oncology, Genetic/Familial High-Risk Assessment: Breast, Ovarian, and Pancreatic, Version 2.2024- September 27, 2023. https://www.nccn.org/guidelines/category_2.

[4] American Cancer Society (2023). Cancer Facts & Figures 2023.

[5] Rosas L.G., Nasrallah C., Park V.T., Vasquez J.J., Duron Y., Garrick O., Hattin R., Cho M., David S.P., Evans J. (2020). Perspectives on Precision Health Among Racial/Ethnic Minority Communities and the Physicians That Serve Them. Ethn. Dis..

[6] Boitano T.K.L., Barrington D.A., Batra S., McGwin G., Turner T.B., Farmer M.B., Brown A.M., Straughn M.J., Leath C.A. (2019). Differences in referral patterns based on race for women at high risk for ovarian cancer in the southeast: Results from a Gynecologic Cancer Risk Assessment Clinic. Gynecol Oncol..

[7] Domchek S.M., Yao S., Chen F., Hu C., Hart S.N., Goldgar D.E., Nathanson K.L., Ambrosone C.B., Haiman C.A., Couch F.J. (2021). Comparison of the Prevalence of Pathogenic Variants in Cancer Susceptibility Genes in Black Women and Non-Hispanic White Women With Breast Cancer in the United States. JAMA Oncol..

[8] Ciuro J., Beyer A., Fritzler J., Jackson N., Ahsan S. (2021). Health Care Disparities and Demand for Expanding Hereditary Breast Cancer Screening Guidelines in African Americans. Clin. Breast. Cancer.

[9] Lau-Min K.S., McCarthy A.M., Nathanson K.L., Domchek S.M. (2023). Nationwide Trends and Determinants of Germline BRCA1/2 Testing in Patients With Breast and Ovarian Cancer. J. Natl. Compr. Cancer Netw..

[10] Rodriguez N.J., Ricker C., Stoffel E.M., Syngal S. (2023). Barriers and Facilitators to Genetic Education, Risk Assessment, and Testing: Considerations on Advancing Equitable Genetics Care. Clin. Gastroenterol. Hepatol..

[11] McCarthy A.M., Bristol M., Domchek S.M., Groeneveld P.W., Kim Y., Motanya U.N., Shea J.A., Armstrong K. (2016). Health Care Segregation, Physician Recommendation, and Racial Disparities in BRCA1/2 Testing Among Women With Breast Cancer. J. Clin. Oncol..

[12] Sutton A.L., He J., Tnner E., Edmonds M.C., Henderson A., Hurtado de Mendoza A., Sheppard V.B. (2019). Understanding Medical Mistrust in Black Women at Risk of BRCA 1/2 Mutations. J. Health Dispar. Res. Pract..

[13] Peterson E.B., Chou W.S., Gaysynsky A., Krakow M., Elrick A., Khoury M.J., Kaphingst K.A. (2018). Communication of cancer-related genetic and genomic information: A landscape analysis of reviews. Transl. Behav. Med..

[14] Dusic E., Bowen D.J., Bennett R., Cain K.C., Theoryn T., Velasquez M., Swisher E., Brant J.M., Shirts B., Wang C. (2022). Socioeconomic Status and Interest in Genetic Testing in a US-Based Sample. Healthcare.

[15] Crenshaw K. (1989). Demarginalizing the Intersection of Race and Sex: A Black Feminist Critique of antidiscrimination Doctrine, Feminist Theory, and Antiracist Politics. Univ. Chic. Legal. Forum..

[16] Estupiñán Fdez de Mesa M., Marcu A., Ream E., Whitaker K.L. (2023). Relationship between intersectionality and cancer inequalities: A scoping review protocol. BMJ Open.

[17] Kelly-Brown J., Palmer Kelly E., Obeng-Gyasi S., Chen J.C., Pawlik T.M. (2022). Intersectionality in cancer care: A systematic review of current research and future directions. Psychooncology.

[18] Sabatello M., Diggs-Yang G., Santiago A., Easter C., Morris K.J., Hollister B.M., Hahn M., Baker K., McCormick A., Greene-Moton E. (2023). The need for an intersectionality framework in precision medicine research. Am. J. Hum. Genet..

[19] Hesse-Biber S., Seven M., Shea H., Heaney M., Dwyer A.A. (2023). Racial and Ethnic Disparities in Genomic Healthcare Utilization, Patient Activation, and Intrafamilial Communication of Risk among Females Tested for BRCA Variants: A Mixed Methods Study. Genes.

[20] Crabtree B.F., Miller W.L., Crabtree B.F., Miller W.L. (1999). Using codes and code manuals: A template organizing style of interpretation. Doing Qualitative Research.

[21] King N., Cassell C., Symon G. (2004). Using templates in the thematic analysis of text. Essential Guide to Qualitative Methods in Organizational Research.

[22] Stake R.E. (1995). The Art of Case Study Research.

[23] Yin R.K. (2018). Case Study Research and Applications: Design and Methods.

[24] Dibble K.E., Donorfio L.K.M., Britner P.A., Bellizzi K.M. (2022). Perceptions and care Recommendations from Previvors: Qualitative analysis of female BRCA1/2 mutation Carriers’ experience with genetic testing and counseling. Gynecol. Oncol. Rep..

[25] Hesse-Biber S., Seven M., Jiang J., Schaik S.V., Dwyer A.A. (2022). Impact of BRCA Status on Reproductive Decision-Making and Self-Concept: A Mixed-Methods Study Informing the Development of Tailored Interventions. Cancers.

[26] Seven M., Shah L.L., Yazici H., Daack-Hirsch S. (2022). From Probands to Relatives: Communication of Genetic Risk for Hereditary Breast-Ovarian Cancer and Its Influence on Subsequent Testing. Cancer Nurs..

[27] Butler E., Collier S., Boland M., Hanhauser Y., Connolly E., Hevey D. (2020). Self-concept and health anxiety relate to psychological outcomes for BRCA1/2 carriers. Psychooncology.

[28] Park S.Y., Kim Y., Kim S., Katapodi M.C. (2023). Informational needs of individuals from families harboring BRCA pathogenic variants: A systematic review and content analysis. Genet. Med..

[29] Coulter A., Oldham J. (2016). Person-centred care: What is it and how do we get there?. Future Hosp. J..

[30] Katapodi M.C., Pedrazzani C., Barnoy S., Dagan E., Fluri M., Jones T., Kim S., Underhill-Blazey M.L., Uveges M.K., Dwyer A.A. (2024). ACCESS: An empirically-based framework developed by the International Nursing CASCADE Consortium to address genomic disparities through the nursing workforce. Front. Genet..

[31] Seven M., Shah L.L., Daack-Hirsch S., Yazici H. (2021). Experiences of BRCA1/2 Gene Mutation-Positive Women With Cancer in Communicating Genetic Risk to Their Relatives. Cancer Nurs..

[32] Mittendorf K.F., Knerr S., Kauffman T.L., Lindberg N.M., Anderson K.P., Feigelson H.S., Gilmore M.J. (2021). Systemic Barriers to Risk-Reducing Interventions for Hereditary Cancer Syndromes: Implications for Health Care Inequities. JCO Precis. Oncol..

[33] Jones T., McCarthy A.M., Kim Y., Armstrong K. (2017). Predictors of BRCA1/2 genetic testing among Black women with breast cancer: A population-based study. Cancer Med..

[34] Williams C.D., Bullard A.J., O’Leary M., Thomas R., Redding T.S., Goldstein K. (2019). Racial/Ethnic Disparities in BRCA Counseling and Testing: A Narrative Review. J. Racial. Ethn. Health Disparities.

[35] Sankar P., Cho M.K., Mountain J. (2007). Race and ethnicity in genetic research. Am. J. Med. Genet. A.

