# Supplementary material for: Intersectionality, BRCA Genetic Testing, and Intrafamilial Communication of Risk: A Qualitative Study
Source: Cancers (Basel). 2024 May 2;16(9):1766. doi: 10.3390/cancers16091766 (PMC11083191; doi:10.3390/cancers16091766)
Supplement: Supplementary file 1 [file cancers-16-01766-s001.zip › cancers-2955786-supplementary.pdf]

**Table S1.** Themes and correspondent category of template analyses of participants experiences		
**Individual level (psychosocial wellbeing)**	**Representative participant quotes:**	**Selected Quotes:**
Barriers to Psychological Wellbeing		***Outcomes of Barriers to Psychosocial Wellbeing:*** *Negatively affecting wellbeing, decision regret, delay in treatment/no treatment*
1. Negative coping styles/Fear<br>2. Lack of social support	1. "Body image is the only thing holding me back. Like you said, it's a risk, right? Because things go wrong all the time. And I think hearing that is terrifying, but I know there are positive and pleasant outcomes as well. But body image I think is big. And I think that's why I'm so much more confident with-I'm willing to have surgery on my ovaries, on my tubes, whatever, because no one's gonna see that or yes, I might have scars but they're going to be in places that aren't that obvious or that or eye catching?" **– ID#17 [33 y.o., Non-Hispanic White Female, $101-125,000/yr.]**<br><br>2. "I started to feel like this level of not necessarily rejection but not being taken seriously. Because you know, I didn't lose my hair, I didn't have to do chemo, I did do a double mastectomy were some people's opinions of that is that that's kind of like extreme for stage 00. But I had a twice within six months. So if people don't see that part, but so what I learned with that is that, you know, I really need to make sure that it's a safe space." **- ID#22 [Female, African American or Black,**	1. "Yeah, I think, for me, the significance of the surgery is very daunting. So the question of, you know, does it make sense? While I'm in this, quote, unquote, healthy age, to have this elective surgery, I think one person could look at it and say, yes, get this prophylactic surgery, while you're healthy, you don't have a cancer diagnosis, and your body can maybe recoup faster. I think I tend to look at it as Oh, my gosh, that's a huge surgery- and If I am healthy why would I put my body through that?" - **ID#17 [Female, Caucasian/White, 33, 101-125k]**<br><br>2. " Because I didn't have that I didn't know all these organizations exist. And so one of my other one of my other missions about for addition to dispelling myths by providing facts, and encouraging preventative health care was also to a form of resources, because I felt like I was on his island all by myself, right. And then when I found an island for breast cancer survivors, it was even further isolation, because people didn't feel that my my cancer was all that serious. So then I learned about this term of our providers as well. And I felt like they must also feel some level of isolation, because people don't take you seriously in a cancer arena."

	48, 151k or more]	ID#22 [Female, African American or Black, 48, 151k or more]
Facilitators of Psychosocial Wellbeing:		***Outcomes of facilitators of psychosocial wellbeing:***<br>*positively affecting wellbeing, decision satisfaction*
1. Positive coping style/personal initiative<br>2. Social support	1. "I think people also don't get it, and I don't understand why they don't get it. It's that, yes you get tested, and these are your options. You can do nothing, but if you do this- and you can have a good life. It may or may not be shortened, but everyone's life may or may not be shortened. Isn't it better to have the knowledge?" **- ID#7 [Female, Caucasian, 57, 51-75k]**<br>2. "Um, the only thing I think I would say is like in terms of finding outside of like my my medical team and my close friends like in terms of trying to find support systems or networks for people who have been through things similar. Like I feel like that I really had to do that all on my own, like my medical team didn't really know anything. I felt like I brought to them." **- ID#4 [Female, Caucasian, Mexican/Mexican American/Chicano, 31, 101-125k]**	1. "But you know, if- having cancer really does make you reevaluate- well, it made me reevaluate my life, end of story. You know, and in a weird way I see it as a blessing because if I didn't- am I still gonna be there, or am I really leading my best life now? You know, I have an amazing life since I blew it up, and part of that whole process, because it is a process, part of it was having the cancer, you know, would I still be doing what I was doing and leading this half-life, or just going, you know "bugger it," I've had cancer, I'll blow everything up. And I did." **- ID#7 [Female, Caucasian, 57, 51-75k]**<br>2. "And I was like, oh, like there's such a great like sort of support system. For women in my situation. And I sort of discovered them all on my own just when I was Googling basically. So I feel like you can take this journey and sort of make it what you want to. So like I was the one who like was very proactive about trying to find outside supports. I sort of wish like my medical team had had something like information on for people like where can you find people who have been through similar injury like similar circumstances or who you can reach out to for emotional support if you need it?" **- ID#4 [Female, Caucasian, Mexican/Mexican American/Chicano, 31, 101-125k]**
**Interpersonal/family level**		

Barriers to Family Communication:		*Outcomes of Barriers:* *Lack of family testing*
1. Family miscommunication<br><br>2. Family cultural environment hindering risk communication<br><br>3. Lack of family history	1. “The cousin that had stage 4 breast cancer was tested but failed somehow. I don't want to say it's stupidity but her (laugh) [...]- Like what the heck, do you know that tells you you have a mutation in your genes, uh, just, you should inform your family. Somehow the disconnect was there so yeah, we found out the hard way.” **-ID#1 [Female, “Mixed” (Caucasian, Mexican/Mexican American/Chicano), 68, >25k]**<br>2. “It's fair to say my father and I have a fairly, uh, strained relationship— Um, but internally within the family it's just kind of a heavy thing that exists between the different generations.” - **ID#3 [Female, “Mixed” (Caucasian, Black, Hispanic), 28, $101-125k]**<br>3. “She like she mentioned she's negative. But she didn't like explain like, the chances of me having it or anything else. Like all the statistics, you know that because I'm my mom probably had it and we have a 50% chance of having it. She never explained that to me. So that's what I was just like, okay, whatever, you know, I didn't pay attention. I didn't research it. Now, I feel like I'm way more informed.” **- ID#25 [Female, Mexican/Mexican American/Chicano, et.]**	1. “So, So she got tested, I didn't know that but when I talked to the daughter and I said you know I’m doing genetic testing and she was like -"oh ya we did that"- ok fine it wouldn’t have made a difference cause within that year frame -I don’t know maybe yeah maybe no- it didn't matter the point is someone didn't tell us [...]Yeah, they failed. They failed to tell the rest of us we had a mutation” **- ID#1 [Female, “Mixed” (Caucasian, Hispanic), 68, >25k]**<br>2. “Um, my paternal, my paternal grandmother, my dad's mom, She refuses to get tested. Um, because she doesn't want to live with the guilt of knowing if she was the one that was positive, or if it was my biological grandfather.” **- ID#3 [Female, “Mixed” (Caucasian, Black, Hispanic), 28, $101-125k]**<br>3. “And I told them, you know, if you have the gene, then you have a 50% chance of passing that gene to your children. And they are so stubborn, and they don't want to get tested [...] That's like, I've tried talking to them and one of my sisters she just says, I'm not ready [...] She doesn't want to know, I think because she feels like she might have it. So I feel like she just doesn't want to cross that bridge. Yeah. Right. Once you know, you can't unring that bell. Right, right. there forever, you know, so it's just like, I think that's what she's scared of my brothers. I think they just don't, they don't think about it. And I try to tell them, you know, guys get breast cancer too and also, is there for prostate cancer.” **- ID#25 [Female, Mexican/Mexican American/Chicano, et.]**

Facilitators of Family Communication:		***Outcomes of Facilitators:*** *Uniform Family Testing*
1. Family communication<br>2. Family cultural environment promoting risk communication<br>3. Knowledge of family history	1. “Yeah. But take into account that my our mother, we are a very big family with a lot of doctors. My father's a doctor, my brother's a doctor, my sister's a doctor, my uncle, my brother. My mother's brother is a doctor. And my mother was a nurse, but not a doctor. I have uh in my mother's and father's side, I have doctors too. So we are very conscious about this. [...] So yeah, we are very, we are not very soft on this.” **- ID#10 [Female, Caucasian (Spanish), 32, 25k or less]**<br>2. “And it's weird, because to me, like, my family is so close, like my sisters, and I talk every day. So the thoughts that they could go like, a year without talking without having had any kind of fight just like not occurring to them to talk be like, bizarre to me.”- **ID#19 [Female, Caucasian (of Central/South American Descent, 38, 151k or more]**<br><br>3. “She, she mentioned having a lineage of breast cancer. And my, my, my, my grandma, she actually died of breast cancer. So my mom she got the cancer when she was quite quite older. So she, as a survivor, she when I mentioned to her I think this can actually be some something related to the gene- something genetical and she would like probably. And then we went ahead on those tests.” **- ID#9 [African American or Black female, 30 yo, 101-125k a year]**	1. “I have one brother and two sisters [...] We were all positive. [...] But my mom was [positive] and the other five brothers, uh four brothers and one from the eight, one we don't know, two are negative, and five are positives, my mom, my auntie, and three boys” **- ID#10 [Female, Caucasian (Spanish), 32, 25k or less]**<br><br>2. “Oh, yeah. I mean, I told my sisters, you know, as soon as I tested positive, and even my mom, and we figured it wasn't from her side, although, interestingly, my cousin on my mom's side that was diagnosed with breast cancer at like, 42, which is pretty young. I mean, like, you know, it's, it's pretty young to have to have breast cancer. So she was tested for BRCA mutations and tested negative. But so just kind of like a fluke thing. But that's, you know, so it's, in a way, like, the fact that I knew, I mean, like, at least she was able, like, at least she knew to get tested.” - **ID#19 [Female, Caucasian (of Central/South American Descent, 38, 151k or more]**<br>3. “So I had to, like, talk to my mom. And she was like to be on the safe side. So we don't let it escalate. We [referring to her three sisters] can actually go ahead and go for a scan and test and find out just to be sure on the safe side and to get early treatment.” **- ID#9 [African American or Black female, 30 yo, 101-125k a year]**

**Healthcare system level**		
Barriers to Receiving Quality health Care:		***Outcomes of Barriers:** delaying of treatment/surgery, lack of control, no continuity of care, high mortality rates*
1. Healthcare professionals creating barriers to emotional and physical wellbeing<br>2. Negative surgical/treatment recovery experiences<br>**3.** Systemic barriers to quality care/care navigation barriers	1. "I think I actually had- I had a rough time with my oncologist. And I don't think we just had a connection the way I wanted it, because I feel like, I came with a lot of questions. Just wanting to, you know, have as much information as possible, right, wanting to feel secure in this doctor's hands, because this was my life I was talking about [...] So that was my second time meeting him, my husband and I. I just got the impression that he felt uncomfortable. Just his body language kind of made me feel like he was uncomfortable around us. So I saw I was like, well, I don't know if he has a lot of African American patients, I'm not sure. But yeah, that is something that crossed my mind." **- ID#18 [Female, African American or Black, 34, 25-50k]**<br>2. "Yeah, he was doing cleavage sparing mastectomies. And what had happened is some women who had done this to, had cancer come back and they've died. And that's why they were investigating it. Unfortunately, he, I have tests further tests. And he'd left an unacceptable amount of tissue behind. But they didn't know how much it left. They didn't know what my risks to breast cancer were. They couldn't give me any information or whatever." **- ID#12 [Female, Caucasian, 56, 25k or less]**<br>**3.** "I think our resources, our financial resources were insufficient. Yes, that's what I feel like that	1. "That, that part, I think, was also really big for me, because it felt like each doctor that I would say to and, you know, everybody is typically has the same kind of response, like, Oh, my goodness, you are so young, and go back and all of these things, and it, it makes me feel even more, I guess, just out of place. It just makes me it made me feel like I just wasn't normal. And I just started kind of avoiding going to the doctors, because I'm like, Oh, I don't I don't want it to be like this every time. So having to tell that over and over. Yeah, yeah, it was really hard." **- ID#18 [Female, African American or Black, 34, 25-50k]**<br>2. "It was just horrendous, really. But then because he'd left tissue behind, they wouldn't give me any HRT, because there was a high risk for breast cancer. So then I've struggled for 10 years, with no HRT, having ridiculous mental health issues, anxiety, and everything. And then in 2020, I finally got HRT, and its [inaudible]. So now I'm doing my menopause party." **- ID#12 [Female, Caucasian, 56, 25k or less]**<br>3. "Yeah, I feel at times it does. I feel there is in many aspects of our life. We are overlooked. Yeah. Because of the color of our skin. And I feel that there are some people who feel as though we don't deserve it. We're not worthy enough to have it. We,

	had a lot to do with it. And, and I'm, I wasn't with my mom the entire time, she went through her ordeal. But I also feel as if though, maybe people may have thought that my mom may not have been intelligent enough to process that information. I don't know. I don't know why. If I even asked him, like, Has anyone talked to you about BRCA? She says, Well, I heard the term you used when I went for the blood test. She said they may have told me but I don't. I don't. I don't. I wouldn't have understood that anyways." - **ID#20 [Female, African American or Black, 47, 76-100k]**	what what we suffered through or what we go through is just our fate and it is what it is." **- ID#20 [Female, African American or Black, 47, 76-100k]**
Facilitators of Quality health Care:		***Outcomes of Facilitators to Quality health Care:*** *Control while making treatment decisions*
1. Healthcare professionals facilitating emotional and physical wellbeing<br>2. Positive surgical/treatment experiences	1. " And so, we ended up going into the exam room, and I met with my doctor. And she was very aggressive. But in a good way, she took the time to get to know me. She asked, you know, she took about 30 to 45 minutes just talking about P011, you know, and then then he got real, you know, she started talking to me about my risk management options. And she was very aggressive. And I was very defensive, very resistant, because it was just, again, another influx of information happening too fast. And but it was what she said to me that really helped me to surrender in a sense." **- ID#11 [Female, African American or Black, 35, 76-100k]**<br>2. "And I'm very, very disapproving [of] private medicine. But, you know, I, in this instance, I	1. " So I'm glad she was the way she was. Because that's what I needed. I needed someone to shed light on how serious this was. And kind of just snapped me out of kind of snapped me in a sense out of like a, like, wake up, you know, out of this, you know this state this, this, this kind of thought process that I was not that she was trying to rescue us from grieving, but she just knew that I was not myself. And she needed to have a sense of urgency in her delivery. That's exactly what she did." **- ID#11 [Female, African American or Black, 35, 76-100k]**<br>2. "And it actually, it made me that they're very angry, or they're upset afterwards, because I thought, for

	just thought, sorry, I'm going for it. [...] I'm going to use my money. I saw somebody private, privately, and I just, I just threw everything I had at it [...] And in fact, I was in hospital within about four weeks."**- ID#5 [Female, Caucasian, 57, 51-75k a year]**	several reasons. One, what a because we have got an NHS and B because of my connections because of my education. Because I'm articulate, because I'm very determined. I actually was able to get exactly what I wanted when I wanted it. And it made me very angry on behalf of people who actually are not in that position." **- ID#5 [Female, Caucasian, 57, 51-75k a year]**
